# Modelling the impact of delaying vaccination against SARS-CoV-2 assuming unlimited vaccine supply

**DOI:** 10.1186/s12976-021-00143-0

**Published:** 2021-07-29

**Authors:** Marcos Amaku, Dimas Tadeu Covas, Francisco Antonio Bezerra Coutinho, Raymundo Soares Azevedo, Eduardo Massad

**Affiliations:** 1 School of Medicine, University of Sao Paulo and LIM01-HCFMUSP, Sao Paulo, Brazil; 2 School of Veterinary Medicine, University of Sao Paulo, Sao Paulo, Brazil; 3 Instituto Butantan, Sao Paulo, Brazil; 4 School of Applied Mathematics, Fundacao Getulio Vargas, Rua Praia de Botafogo 190, Rio de Janeiro, RJ CEP 22250-900 Brazil; 5 London School of Hygiene and Tropical Medicine, Keppel St., London, WC1E 7HT UK

**Keywords:** COVID-19, SARS-CoV-2, Mathematical Models, Vaccines

## Abstract

**Background:**

At the moment we have more than 177 million cases and 3.8 million deaths (as of June 2021) around the world and vaccination represents the only hope to control the pandemic. Imperfections in planning vaccine acquisition and difficulties in implementing distribution among the population, however, have hampered the control of the virus so far.

**Methods:**

We propose a new mathematical model to estimate the impact of vaccination delay against the 2019 coronavirus disease (COVID-19) on the number of cases and deaths due to the disease in Brazil. We apply the model to Brazil as a whole and to the State of Sao Paulo, the most affected by COVID-19 in Brazil. We simulated the model for the populations of the State of Sao Paulo and Brazil as a whole, varying the scenarios related to vaccine efficacy and compliance from the populations.

**Results:**

The model projects that, in the absence of vaccination, almost 170 thousand deaths and more than 350 thousand deaths will occur by the end of 2021 for Sao Paulo and Brazil, respectively. If in contrast, Sao Paulo and Brazil had enough vaccine supply and so started a vaccination campaign in January with the maximum vaccination rate, compliance and efficacy, they could have averted more than 112 thousand deaths and 127 thousand deaths, respectively. In addition, for each month of delay the number of deaths increases monotonically in a logarithmic fashion, for both the State of Sao Paulo and Brazil as a whole.

**Conclusions:**

Our model shows that the current delay in the vaccination schedules that is observed in many countries has serious consequences in terms of mortality by the disease and should serve as an alert to health authorities to speed the process up such that the highest number of people to be immunized is reached in the shortest period of time.

## Introduction

As the world struggles to implement vaccination schemes against the Severe acute respiratory syndrome with coronavirus 2 (SARS-CoV-2), limited production of doses, imperfections in planning vaccine acquisition and difficulties in implementing distribution among the population, have hampered the control of the virus so far [1]. As of 18 February 2021, 188 million people have been vaccinated around the world, which represents less than 3% of the total. In Brazil, the total number of vaccinated people so far is around 2.5% of the target population [2]. The world vaccination rate currently is less than 4 million doses per day, a very small rate [3]. So, it is no surprise that vaccination by itself has had so far little effect on the number of cases and deaths that continues to soar in many countries. At the moment we have more than 177 million cases and 3.8 million deaths (as of June 2021) around the world [4].

Although it has been possible to bring some previous pandemics under control without pharmaceutical interventions, this has not been possible with COVID-19 and there is a growing body of evidences that this will not be the case with the vaccines against COVID-19 [5–8].

Immediately after the emergence of SARS-CoV-2 in China, many laboratories around the world started the development of more than 100 types of different vaccines, reducing in less than one year the usual time frame of new vaccines development and testing, which normally would be around ten years, a remarkable effort [6, 9].

There is a wide range of covid-19 vaccines being developed [10]. As of june 2021, there are 122 vaccine candidates in 361 clinical trials and 17 vaccines approved by at least one country [10].Of these, 119 vaccines are in the pipeline, of which 35 are in Phase 3 of clinical trials (four have already completed this phase) and 49 in Phase 2 [10]. In the United States of America (USA), three vaccines completed Phase 3 trials, namely, Moderna, Pfizer, and Oxford-AstraZeneca [10].

However, in order to have significant impact on the course of the pandemic, safe and effective vaccines have to emerge in less time that it would take the affected populations to reach natural herd immunity because to wait to have natural herd immunity would result in millions of deaths. Therefore, an unprecedented time-schedule to roll out any effective vaccine is urgently needed. Nevertheless, in many countries the vaccination is limited to certain individual groups and the distribution of enough doses for these individuals is very slow. We have at the moment 2.5 billiion doses applied around the world [11].

Mathematical models have played a key role in helping understanding of COVID-19 dynamics as well as in determining the best decisions about mitigation strategies [12]. In this sense, models remain essential tools for evidence synthesis, planning and forecasting and decision analysis for COVID-9 control and policymaking [12].

In the context of vaccination, the limited initial supply of COVID-19 vaccines raises the question on how to prioritize doses [13]. In addition, there is a conflict between infection and vaccination with infections still growing exponentially in many countries around the world, whereas vaccination rates are inherently restricted by supply and logistics [14]. There is, therefore, a need for a model-informed approach to quantify the impact of COVID-19 vaccination on the course of the epidemic [13].

Brazil has accumulated almost 18 million cases and more than 490 thousand deaths at the time of writing (18 June 2021) [15]. The state of Sao Paulo, the most populous in Brazil reported more than 3 million cases and 120 thousand deaths so far [16]. Notwithstanding the fact that Brazil is the third country with the highest number of cases and second with the highest number of deaths in the world, four vaccines, Coronavac, Oxford-AstraZeneca, Pfiser and Jansen have been licensed. Currently, just above 11% of the target population have received two doses of one of the vaccines [17].

This paper proposes a new model to estimate the impact of vaccination delay against COVID-19 on the number of cases and deaths by the disease in Brazil. We apply the model to Brazil as a whole and to the State of Sao Paulo, the state most affected by COVID-19 in Brazil. This work is a theoretical exercise because it assumes that throughout the pandemic there is enough vaccine, which is not realistic for the majority of countries.

### The model

The model is an extension of the one presented in [18] and has the following variables:

1. *Susceptible* individuals, denoted *S*(*t*), which can either be vaccinated with rate *v*, or acquire the infection with rate β (per infected individual). Susceptible are born with rate Λ and die by other causes with rate μ;
2. *Vaccinated* individuals, denoted *V*(*t*), which are transferred from the susceptible state with the per capita vaccination rate *v*. The vaccine is assumed to have efficacy *q* and a fraction *w* of the susceptible individuals comply with the vaccination policy. Vaccinated individuals die by other causes with rate μ;
3. *Failure* to be immunized individuals, denoted *FV*(*t*). A fraction (1-*q*) of vaccinated individuals fail to be immunized (that is, not completely immunized after vaccination), and can either acquire the infection with the same rate β as those non-vaccinated susceptible individuals or die by other causes with rate μ;
4. *Exposed* individuals, denoted *E*(*t*), are those individuals who acquired the infection but are still in the incubation period that precedes either the overtly diseased patients or the asymptomatic individuals (see below). Exposed individuals can either progress to an asymptomatic stage with rate δ_A_, or to full-blown COVID-19 patients with rate δ_I_ or die by other causes with rate μ. A fraction *p*_E_ of those exposed are infective to susceptible individuals;
5. *Asymptomatic* (or pauci-symptomatic) individuals, denoted *A*(*t*), who progressed from the exposed and are, therefore, infected with SARS-CoV-2 but show no or very few symptoms. Asymptomatic individuals can either die by natural causes or by the infection, with rates μ and α_A_, respectively, or recover from the infection with rate γ_A_. A fraction *p*_A_ of these asymptomatic individuals are infective to susceptible;
6. *Infective* individuals, denoted *I*(*t*), are those individuals infected with SARS-CoV-2 and who show the characteristic clinical signs and symptoms of COVID-19. Infective individuals can either die by natural causes or by the infection, with rates μ and α_*I*_, respectively, or recover from the infection with rate γ_I_, or progress to hospitalized (*H*(*t*)) or critically ill stages (*G*(*t*)) (see below) with rates σ_H_ and σ_G_, respectively;
7. *Hospitalized* individuals, denoted *H*(*t*), are individuals with full-blown COVID-19 but who do not require Intensive Care Unit support. These individuals can die by natural causes or by the infection, with rates μ and α_H_, respectively, or recover from the infection with rate γ_H_;
8. *Gravely ill* patients, denoted *G*(*t*), are seriously ill patients requiring Intensive Care respiratory support. These individuals can die by natural causes or by the infection, with rates μ and α_H_, respectively, or recover from the infection with rate γ_G_; and finally
9. *Recovered* individuals, denoted *R*(*t*), are those individuals who have recovered from the infection. They can die by natural causes with rate μ.

**Fig. 1:**
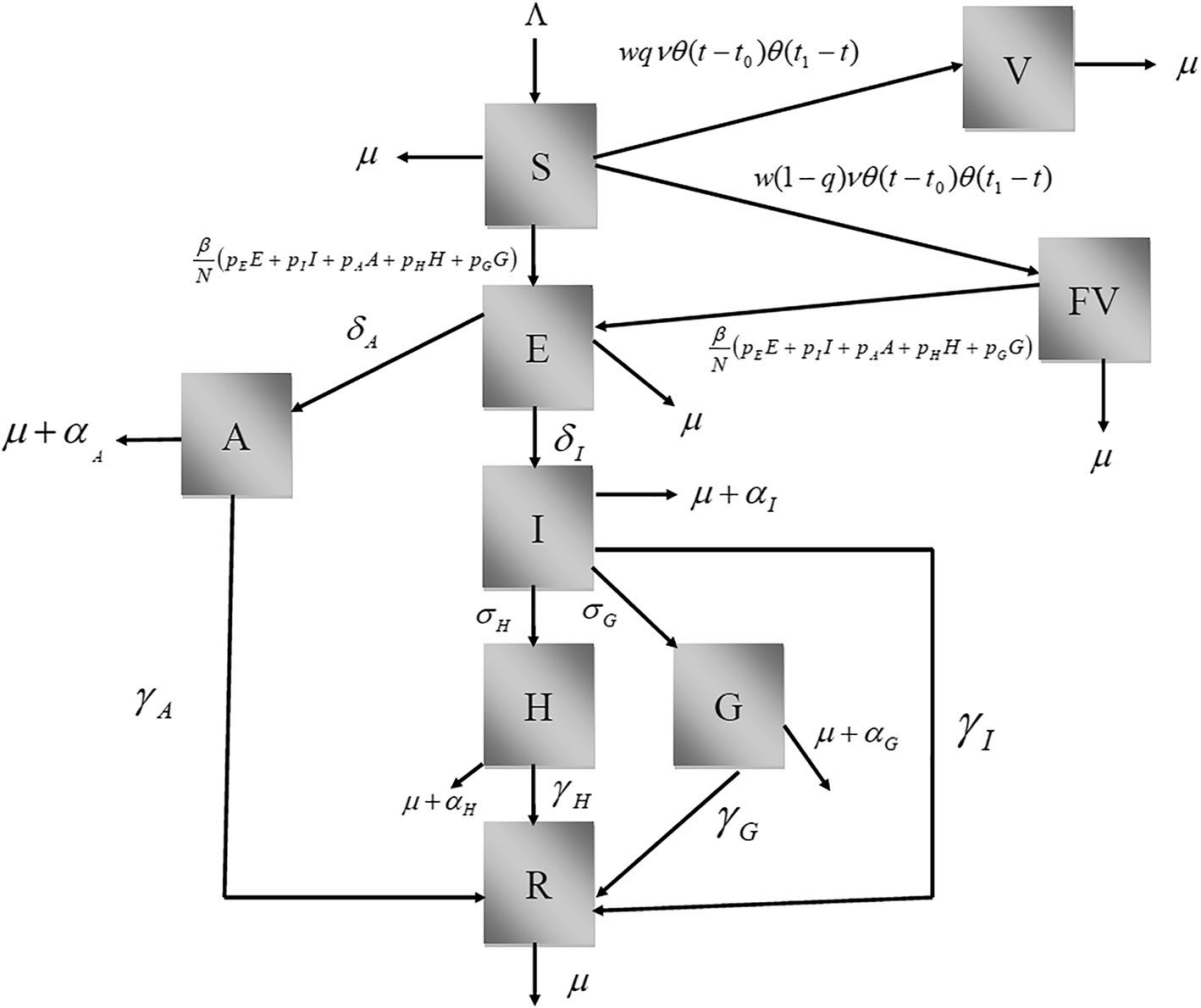
Diagram showing the model’s stages and transitions. The figure shows a block diagram with all the model’s states, namely, susceptible (S), vaccinated (V), vaccination failure (FV), exposed to the virus (E), which here is assumed to mean infected but not yet symptomatic, yet potentially still infectious, asymptomatic (A), infected and infectious (I), hospitalized (H), ICU patients (G) and recovered from infection (R). Transitions from one compartment to other are represented as rates, as described in the main text

Figure 1 shows a diagram with the model’s stages and transitions.

The model is described by the following set of equations:

1 $$\begin{array}{c}\frac{dS\left(t\right)}{dt}=-\frac{\beta }{N}S\left(t\right)\left[{p}_{E}E\left(t\right)+{p}_{A}A\left(t\right)+{p}_{I}I\left(t\right)+{p}_{H}H\left(t\right)+{p}_{G}G\left(t\right)\right]-\nu wS\left(t\right)\theta \left(t-{t}_{0}\right)\theta \left({t}_{1}-t\right)+\Lambda \left(\mathrm{t}\right)-\mu S\left(t\right)\\ \frac{dE(t)}{dt}=\frac{\beta }{N}\left[S\left(t\right)+FV(t)\right]\left[{p}_{E}E\left(t\right)+{p}_{A}A\left(t\right)+{p}_{I}I\left(t\right)+{p}_{H}H\left(t\right)+{p}_{G}G\left(t\right)\right]-\left({\delta }_{A}+{\delta }_{I}+\mu \right)E(t)\\ \begin{array}{c}\frac{dA(t)}{dt}={\delta }_{A}E\left(t\right)-\left({\gamma }_{A}+{\alpha }_{A}+\mu \right)A\left(t\right)\\ \frac{dI(t)}{dt}={\delta }_{I}E\left(t\right)-\left({\sigma }_{H}+{\sigma }_{G}+{\gamma }_{I}+{\alpha }_{I}+\mu \right)I\left(t\right)\\ \begin{array}{c}\frac{dH(t)}{dt}={\sigma }_{H}I\left(t\right)-\left({\gamma }_{H}+{\alpha }_{H}+\mu \right)H\left(t\right)\mu \\ \frac{dG(t)}{dt}={\sigma }_{G}I\left(t\right)-\left({\gamma }_{G}+{\alpha }_{G}+\mu \right)G\left(t\right)\\ \begin{array}{c}\frac{dR(t)}{dt}={\gamma }_{A}A\left(t\right)+{\gamma }_{I}I\left(t\right)+{\gamma }_{H}H\left(t\right)+{\gamma }_{G}G\left(t\right)-\mu R\left(t\right)\\ \frac{dV(t)}{dt}=\left[\nu qwS\left(t\right)-\mu V\left(t\right)\right]\theta \left(t-{t}_{0}\right)\theta \left({t}_{1}-t\right)\\ \begin{array}{c}\frac{dFV(t)}{dt}=\left\{\nu \left(1-q\right)wS\left(t\right)-\frac{\beta }{N}FV\left(t\right)\left[{p}_{E}E\left(t\right)+{p}_{A}A\left(t\right)+{p}_{I}I\left(t\right)+{p}_{H}H\left(t\right)+{p}_{G}G\left(t\right)\right] -\mu FV(t)\right\}\theta \left(t-{t}_{0}\right)\theta \left({t}_{1}-t\right)\\ N\left(t\right)=S\left(t\right)+E\left(t\right)+A\left(t\right)+I\left(t\right)+H\left(t\right)+G\left(t\right)+R\left(t\right)+V\left(t\right)+FV\left(t\right)\\\Lambda \left(\mathrm{t}\right)=\mu N\left(t\right)\end{array}\end{array}\end{array}\end{array}\end{array}$$

In the model, the θ function is the Heaviside step-function, included to simulate different times of starting vaccination.

The incidence, *Inc*(*t*), is given by:

2 $$Inc\left(t\right)=\beta S\left(t\right)+FV(t)\theta \left(t-{t}_{0}\right)\theta \left({t}_{1}-t\right)\left[{p}_{E}E\left(t\right)+{p}_{A}A\left(t\right)+{p}_{I}I\left(t\right)+{p}_{H}H\left(t\right)+{p}_{G}G\left(t\right)\right]$$

The total number of cases, *Cases*, is given by:

3 $$Cases={\int }_{0}^{\infty }\frac{\beta }{N}\left\{\left[S\left(t\right)+FV(t)\theta \left(t-{t}_{0}\right)\theta \left({t}_{1}-t\right)\right]\left[{p}_{E}E\left(t\right)+{p}_{A}A\left(t\right)+{p}_{I}I\left(t\right)+{p}_{H}H\left(t\right) +{p}_{G}G\left(t\right)\right]\right\}dt$$

The total number of deaths, *Deaths* due to COVID-19,is given by:

4 $$Deaths={\int }_{0}^{\infty }\left[{\alpha }_{A}A\left(t\right)+{\alpha }_{I}I\left(t\right)+{\alpha }_{H}H\left(t\right)+{\alpha }_{G}G(t)\right]dt$$

The total number of vaccinated individuals, *Vaccinated*, is given by:

5 $$Vaccinated={\int }_{0}^{\infty }\left[\nu wS\left(t\right)\theta \left(t-{t}_{0}\right)\theta \left({t}_{1}-t\right)\right]dt$$

In Table 1 we show the parameters used for the simulation of model (1) for Brazil and the State of São Paulo.

**Table 1:** Parameters used in the model for the State of São Paulo and Brazil

Parameter	Description	Value	
		**São Paulo**	**Brazil**
$\beta (t)$	Potentially infective contact rate	Fitted (changes over time)	
$p_{E}$	Infectivity of exposed individuals	0.4^a^	0.4^a^
$p_{I}$	Infectivity of symptomatic individuals	1.0^a^	1.0^a^
$p_{A}$	Infectivity of asymptomatic individuals	1/3^a^	1/3^a^
$p_{H}$	Infectivity of hospitalized individuals	0.01^a^	0.01^a^
$p_{G}$	Infectivity of ICU patients	0.01^a^	0.01^a^
$\mu$	Natural mortality rate (life expectancy of 70 years)	3.91 × 10^–5^ days^−1^ ^a^	3.91 × 10^–5^ days^−1^ ^a^
$\delta_{I}$	Rate of evolution from exposed to infected	1/2 day^−1^ ^a^	1/2 day^−1^ ^a^
$\delta_{A}$	Rate of evolution from exposed to asymptomatic	0.874 day^−1^ ^b^	0.366 day^−1^ ^b^
$\gamma_{I}$	Rate of recovery from infected	1/3 day^−1^ ^a^	1/3 day^−1^ ^a^
$\gamma_{A}$	Rate of recovery from asymptomatic	1/14 day^−1^ ^a^	1/14 day^−1^ ^a^
$\gamma_{H}$	Rate of recovery from hospitalized	1/10 day^−1^ ^a^	1/10 day^−1^ ^a^
$\gamma_{G}$	Rate of recovery from ICU	0.05 day^−1^ ^b^	0.0556 day^−1^ ^b^
$\alpha_{I}$	Disease-induced mortality rate for infected individuals	5 × 10^–4^ day^−1^ ^a^	3 × 10^–4^ day^−1^ ^a^
$\alpha_{A}$	Disease-induced mortality rate for asymptomatic individuals	0 ^a^	0 ^a^
$\alpha_{H}$	Disease-induced mortality rate for hospitalized individuals	10^–4^ day^−1^ ^b^	5.56 × 10^–4^ day^−1^ ^b^
$\alpha_{G}$	Disease-induced mortality rate for ICU patients	Fitted (changes over time)	
$\sigma_{H}$	Hospitalization rate	0.12 day^−1^ ^b^	0.0518 day^−1^ ^b^
$\sigma_{G}$	ICU admission rate	Fitted (changes over time)	
$\nu$	Vaccination rate	Variable (from 5 × 10^–3^ days^−1^ to 10^–1^ days^−1^)	
$q$	Vaccination efficacy	Variable	
$w$	Adherence to the vaccination campaign	Variable	
$K(t)$	Notification ratio	Fitted (changes over time)	
$\Lambda (t)$	Birth rate	Changes over time	

^a^assumed; ^b^fitted

**Fig. 2:**
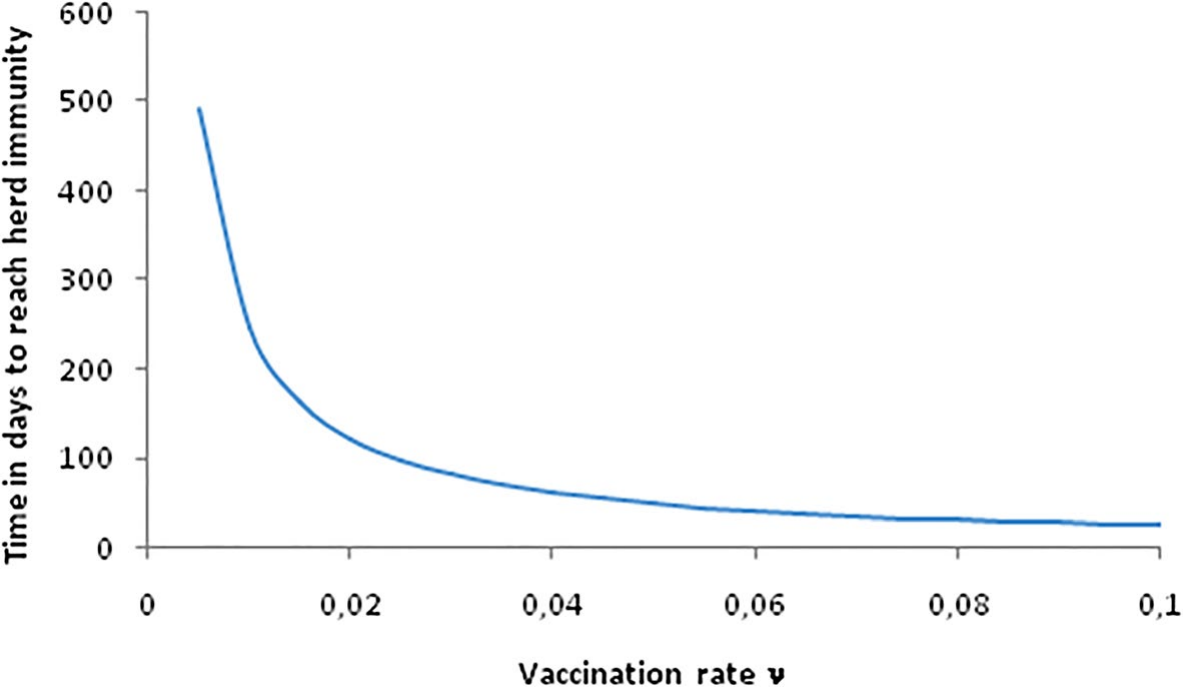
Time in days taken to reach herd immunity as a function of vaccination rates as used in the simulation of the model

In Fig. 2 we show the time that it would take to reach herd immunity (70% of the population, i.e. *R*_*0*_ = 3.3) as a function of the vaccination rate, *v*. This is calculated, approximately (neglecting mortality) by solving the system of equations:

6a $$\frac{dS}{dt}=-\nu wqS$$

6b $$\frac{dV}{dt}=\nu wqS$$

We note that this is a significant simplification, and is not intended to be a realistic representation of vaccination in the real world. Although in reality the number of vaccinated hosts typically depends on the number of available doses per day, for the purpose of this model we instead assume that the net vaccination rate is proportional to the number of individuals who are currently susceptible and unvaccinated. We also do not consider multiple doses, as are often required for COVID-19 vaccination.

## Results

We simulated model (1) with the parameters as in Table 1 for the two populations of the State of Sao Paulo and Brazil as a whole, varying the scenarios related to vaccine efficacy and compliance from the populations. We simulated vaccine efficacy (defined by the effect of the vaccine at preventing infections) with values of 50%, 70% and 90% and compliance with values of 50%, 70% and 80%. As mentioned above, we simulated the scenarios for Brazil as a whole and for the State of São Paulo. As shown in Table 1, we have chosen values of vaccination rates that varied from 0.005 days^−1^ to 0.1 days^−1^.

Below we show the results of the numerical simulations of the model.

**Fig. 3:**
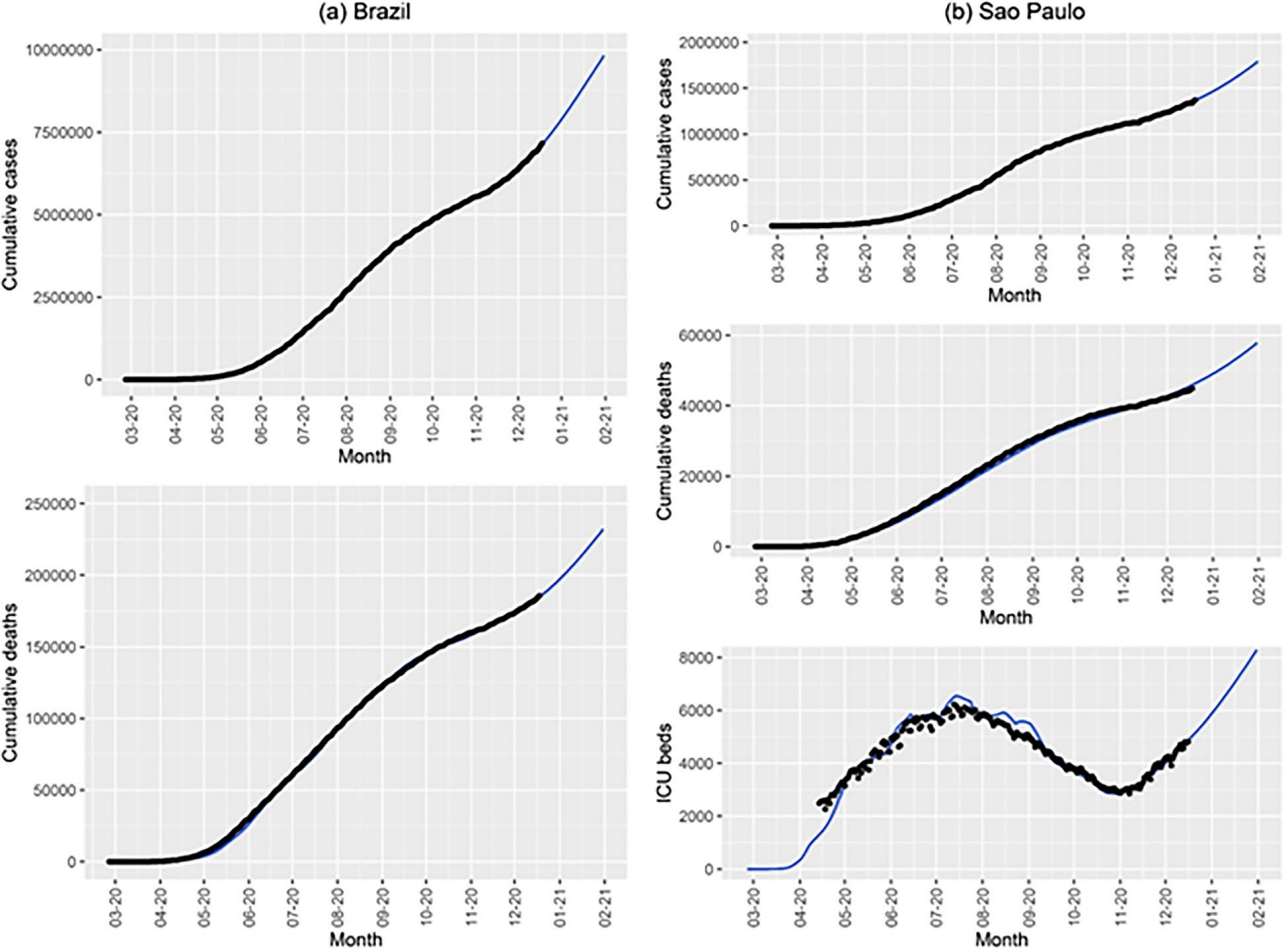
Cumulative number of reported cases and deaths in (**a**) Brazil (black dots) and cases, deaths, and number of ICU patients in (**b**) the State of Sao Paulo. The blue lines correspond to the fitted models

We fitted the model parameters simultaneously to the data of cumulative number of reported cases and deaths (Fig. 3(a)) for Brazil and to the data of cumulative number of reported cases, deaths and the number of intensive care units (ICU) patients (Fig. 3(b)) for the State of Sao Paulo until December 18, 2020. The fitting procedure is described in [18–20].

**Fig. 4:**
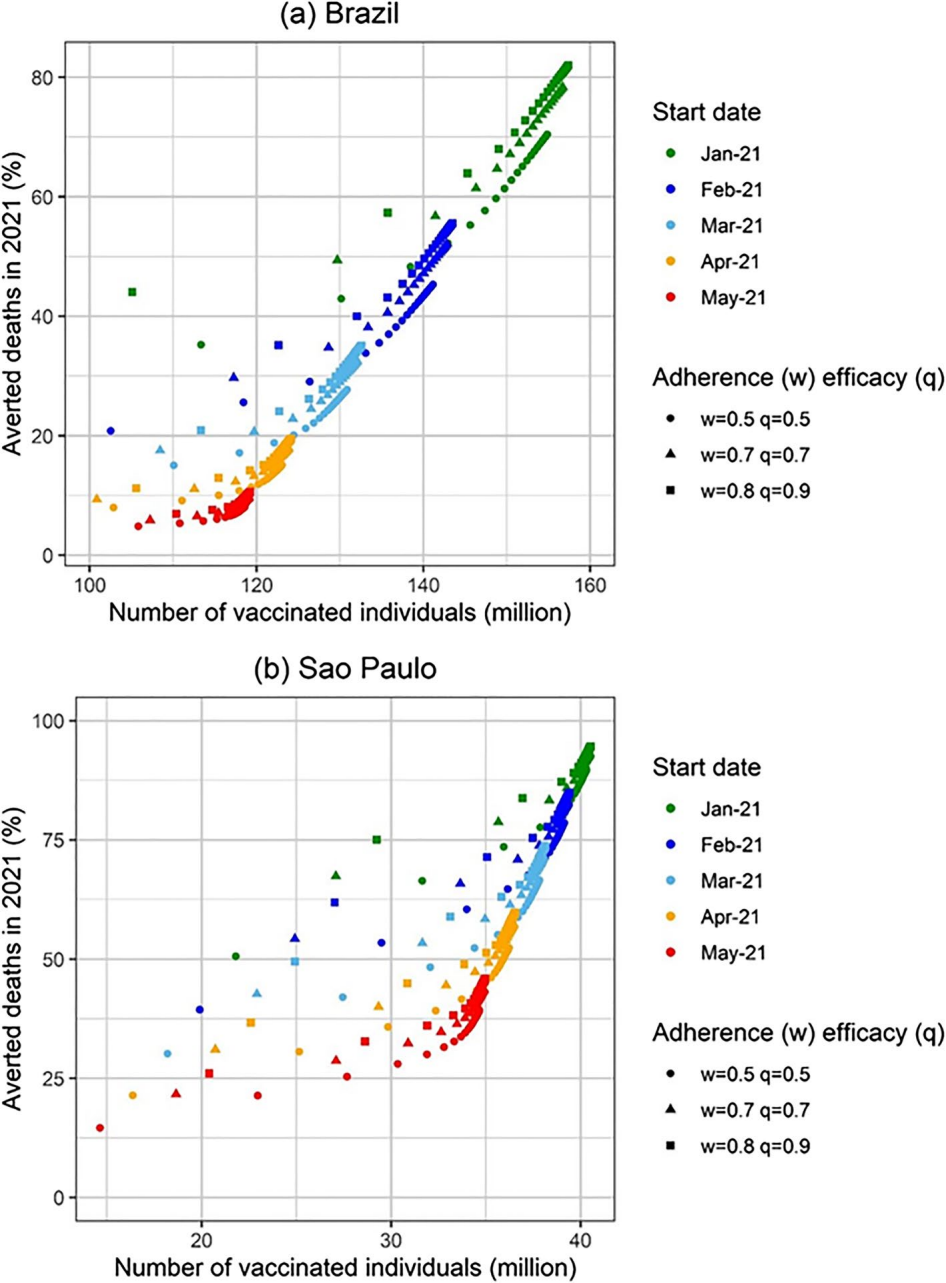
Percentage of averted deaths in 2021 in (**a**) Brazil and (**b**) the State of Sao Paulo as a function of the number of vaccinated individuals for different start dates for the vaccination campaign. Three different combinations of vaccination adherence (w) and vaccine efficacy (q) were considered: w = 0.8 and q = 0.9 (best-case scenario), w = 0.7 and q = 0.7 (baseline scenario) and w = 0.5 and q = 0.5 (worst-case scenario)

In Fig. 4 we show the percentage of averted deaths until 31^st^ December 2021 for several scenarios simulated and for mass vaccination starting on January 21^st^, February 21^st^, March 21^st^, April 21^st^ and May 21^st^, for 3 combinations of population vaccination compliance and vaccine effectiveness.

It can be noted from Fig. 4(a) that if Brazil had started a mass vaccination campaign on January 21^st^ with the maximum compliance of 80%, a vaccine that is 90% efficacious, and a high vaccination rate, 80% of the expected deaths until December 31^st^ would be averted.

**Fig. 5:**
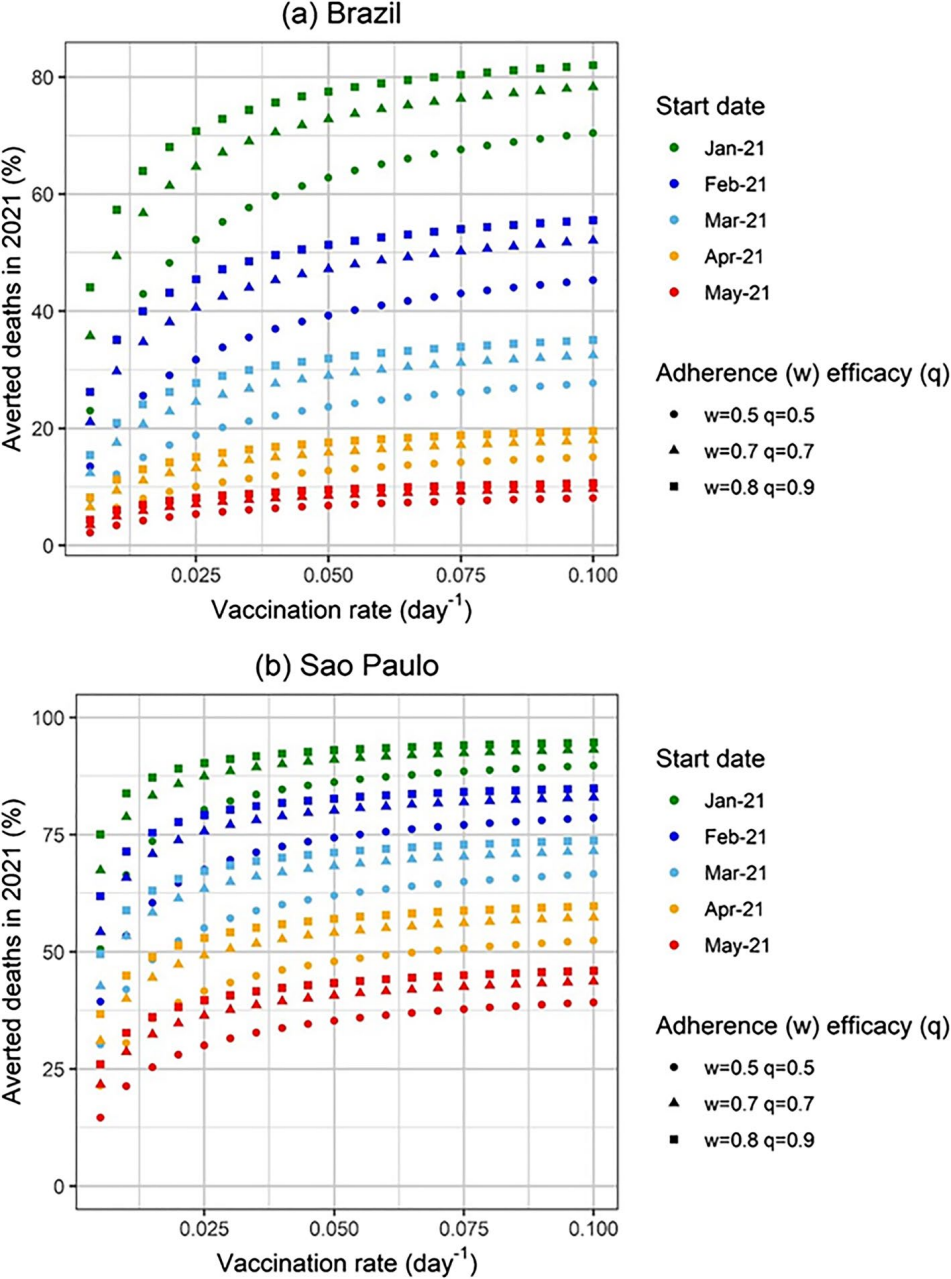
Percentage of averted deaths in 2021 in (**a**) Brazil and (**b**) the State of Sao Paulo as a function of the vaccination rate for different start dates for the vaccination campaign. Three different combinations of vaccination adherence (w) and vaccine efficacy (q) were considered: w = 0.8 and q = 0.9 (best-case scenario), w = 0.7 and q = 0.7 (baseline scenario) and w = 0.5 and q = 0.5 (worst-case scenario)

This result can also be seen in Fig. 5 in which we show the percentage of averted deaths until the end of the year as a function of the vaccination rate for vaccination starting from January until May, with several vaccination scenarios, varying compliance, efficacy and date of the starting of the campaign.

**Fig. 6:**
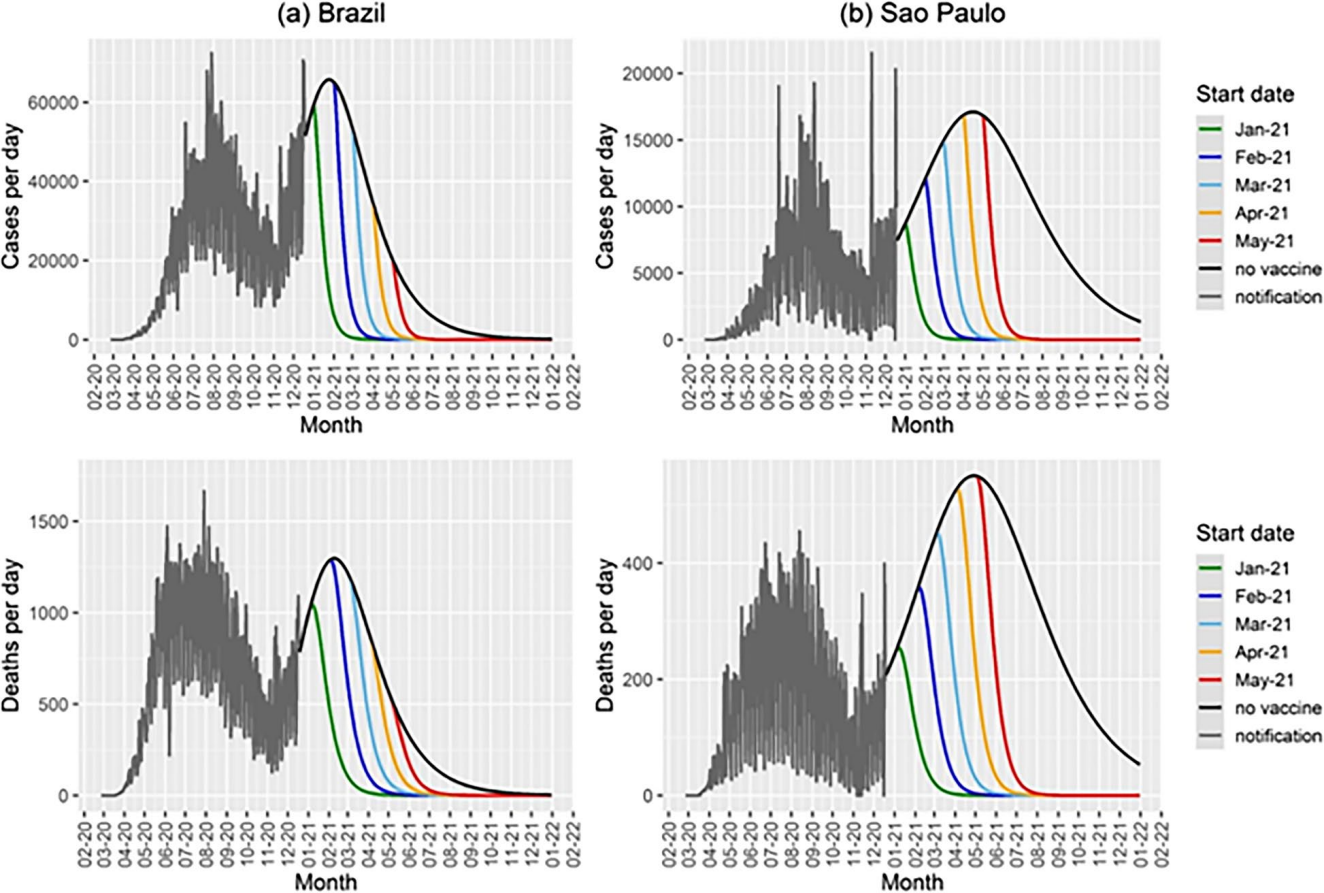
Cases and deaths per day (**b**) as a function of time for different start dates for the vaccination campaign in (**a**) Brazil and (**b**) the State of Sao Paulo for a vaccination rate of 0.05 day^−1^, and the results for the model with no vaccination (black lines). The notification data until December 18^th^ 2020 are shown in gray

In Fig. 6 we show the model’s projection in terms of daily new cases and deaths for an intermediate vaccination rate and for the 5 different starting dates for the vaccination campaign. In the figure, we show the simulation with a vaccination rate of 0.05 days^−1^. This implies that with this rate the country would take approximately one year to reach the herd immunity, assumed to be 70% (i.e., a basic reproduction number equal to 3.3) of the population. In addition, this vaccination rate means an average 544 thousand vaccinations per day in Brazil, provided that there would be enough vaccine supply to this schedule.

In Fig. 6 it is possible to observe the projected number of cases and deaths in the absence of vaccination and with the campaign beginning in January, February, March, April or May.

**Fig. 7:**
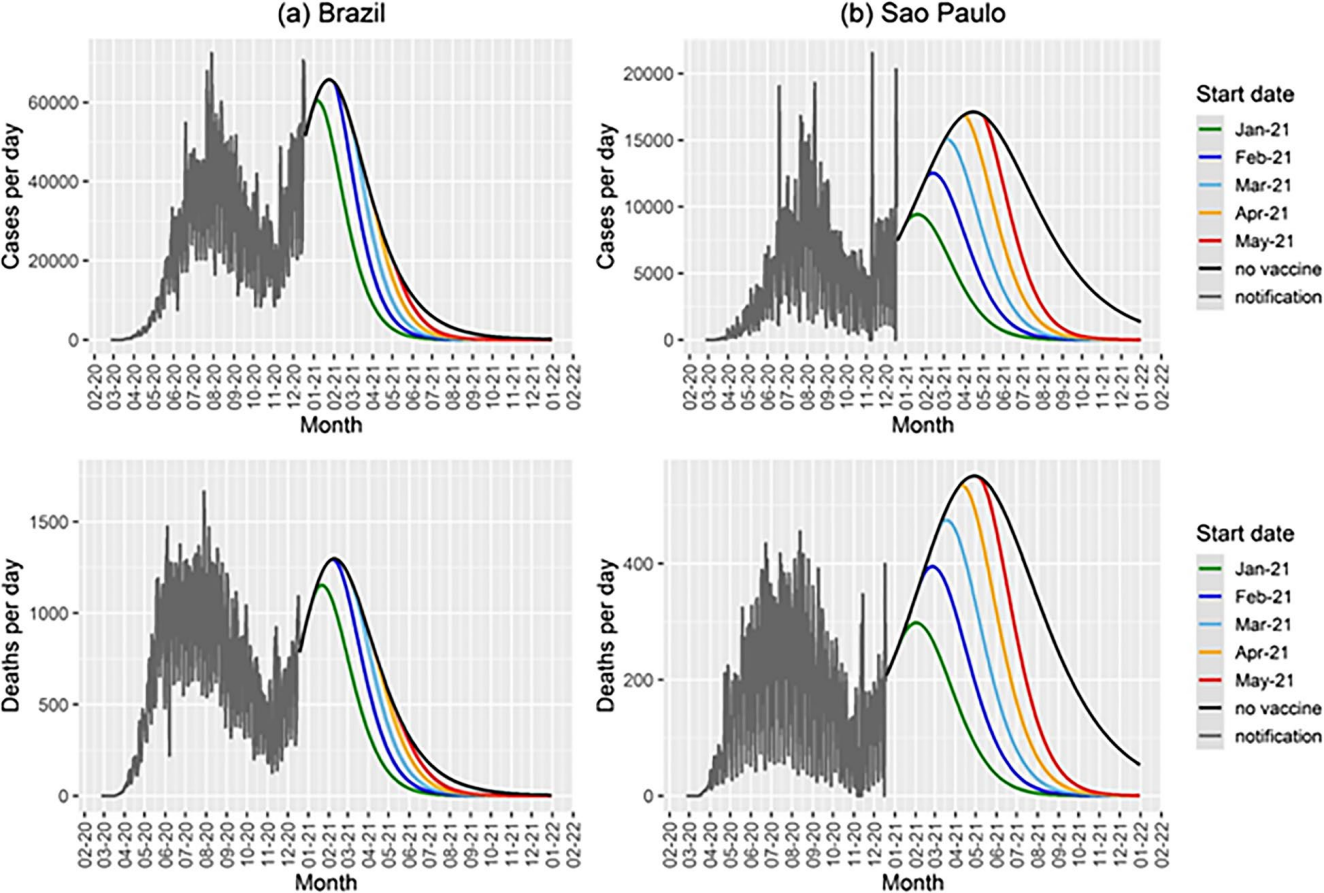
Cases and deaths per day as a function of time for different start dates for the vaccination campaign in (**a**) Brazil and (**b**) the State of Sao Paulofor a vaccination rate of 0.005 day^−1^, and the results for the model with no vaccination (black lines). The notification data until December 18^th^ 2020 are shown in gray

In Fig. 7 we show the same simulated scenarios as in Fig. 6 with a vaccination rate ten times lower.

In Tables 2 and 3 we summarize our main results for one particular scenario maximizing the vaccination rate, compliance of the population (80%) and vaccine efficacy (90%), that is, what we should expect in the optimal condition. The simulations are for Sao Paulo and Brazil as a whole.

**Table 2:** Total number of expected deaths by 31 December 2021 for an optimized vaccination

	**Total deaths until 31 December 2021**	
	**São Paulo**	**Brazil**
No Vaccination	168,290	352,931
Beginning vaccination on 21^st^ January 2021	55,489	225,289
Beginning vaccination on 21^st^ February 2021	67,118	266,474
Beginning vaccination on 21^st^ March 2021	80,384	298,341
Beginning vaccination on 21^st^ April 2021	97,093	322,580
Beginning vaccination on 21^st^ May 2021	113,545	336,475

**Table 3:** Number of additional deaths due to vaccination delay in the optimum scenario

	**Expected number of deaths due to vaccination** **delay until 31 December 2021**	
	**Sao Paulo**	**Brazil**
1 month delay	11,629	41,185
2 months delay	24,895	73,052
3 months delay	41,604	97,291
4 months delay	58,056	111,186

It can be noted from Table 2 that, in the absence of vaccination, the model projects almost 170 thousand deaths and more than 350 thousand deaths until the end of 2021 for Sao Paulo and Brazil, respectively. If in contrast, Sao Paulo and Brazil had enough vaccine supply and so started a vaccination campaign in January with the maximum vaccination rate, compliance and efficacy, they could have averted more than 112 thousand deaths and 127 thousand deaths, respectively.

In Table 3 we show the number of additional deaths attributable to vaccination delay. It can be seen that for each month of delay the number of deaths increases monotonically (in a logarithmic fashion) for both the State of Sao Paulo and Brazil as a whole.

## Discussion

In this paper we present a theoretical exercise, represented by a model intended to estimate the impact of (perhaps inevitable) delays in starting vaccination against SARS-CoV-2, illustrated with the epidemic situation in Brazil and in the State of Sao Paulo. The model parameters are calibrated from reports of daily COVID-19 infections, as well as published reports, despite the simplifications made, our model, it reproduces the real data with remarkable accuracy. Our results demonstrate that, both for Brazil as a whole and for the State of Sao Paulo, for each month of delaying the starting of vaccination, the number of additional deaths due to COVID-19 is staggering high.

We assumed vaccination rates that simulate immunization of up to 70% of the whole country in 9 months, which may seem unfeasible but Brazil has a long tradition of mass vaccination campaigns [21], managing to immunize more than 20 million people in a single day [22]. Therefore, the maximum vaccination scenario would be a real possibility, given the country’s experience of mass vaccination schedules adopted in the past. However, due to difficulties in vaccine acquisition, the number of available doses so far has been very low indeed [23]. At the time of writing, Brazil has vaccinated slightly above 2% of its population, way below the target of at least 70% to achieve the assumed herd immunity level.

The model has some important limitations worth mentioning, the most important is perhaps that it does not consider age-dependence in incidence of the infection and in the mortality rates. However, the model was intended to simulate a mass vaccination campaign that would include all age strata in a relatively short period of time. In addition, we considered only the original variant of the virus, which means that our results represent a lower bound in the number of cases and deaths due to vaccination delay. The current scenario of the pandemic, in which new variants of SARS-CoV-2 are emerging in some countries [24, 25] should be considered in the simulation of future vaccination models.

Another important limitation is that in the model only having one vaccine dose is considered, however many of the current vaccines require two doses. This assumption that everyone just requires one dose to be fully immunized was done to simplify the calculations, and despite the fact that many vaccines require two doses, this would not significantly change the results of the model. In future models we intend to apply a more realistic scenario with two doses of the vaccine.

Reported cases, deaths and number of occupied ICU beds were fitted simultaneously for Sao Paulo, while only cases and deaths were fitted for Brazil, because a dataset with the daily number of ICU patients was only available for Sao Paulo. As a result, the accuracy of the parameter estimates related to hospitalized patients in Brazil may have been affected.

Finally, we should note that the vaccination rate (scaled by the size of the host population) currently applied in Brazil is estimated to be 10 times less than the one applied, for instance in Israel. With the current vaccination rates it will take more than a year to reach herd immunity in Brazil, even if the effect of the vaccine does not wane over time.

## Conclusion

In conclusion, our model shows that the current delay in the vaccination schedules, that is observed in many countries, has serious consequences in terms of mortality by the disease and should serve as an alert to health authorities to speed the process up such that the highest number of people to be immunized is reached in the shortest period of time.

## Data Availability

The dataset used and analysed during the current study are available from the corresponding author on reasonable request.

## Abbreviations

COVID-19: Coronavirus induced disease 2019
SARS-CoV-2: Severe acute respiratory syndrome by the coronavirus 2
USA: United States of America
*S*(*t*): Susceptible individuals
*V*(*t*): Vaccinated individuals
*FV*(*t*): Failure to be immunized individuals
*E*(*t*): Exposed individuals
*A*(*t*): Asymptomatic individuals
*I*(*t)*: Infective individuals
*H*(*t*): Hospitalized individuals
*G*(*t*): Grave individuals
*R*(*t*): Recovered individuals
*R*_*0*_: Basic Reproduction Number
ICU: Intensive Care Unit
